# The central arterial stiffness parameters in decompensated versus compensated states of heart failure: a paired comparative cohort study

**DOI:** 10.1186/s43044-021-00236-8

**Published:** 2022-01-03

**Authors:** Ahmed El Fol, Waleed Ammar, Yasser Sharaf, Ghada Youssef

**Affiliations:** Cardiovascular Department, Faculty of Medicine, Cairo University, Cairo, Egypt

**Keywords:** Heart failure, Pulse wave analysis, Vascular stiffness

## Abstract

**Background:**

Arterial stiffness is strongly linked to the pathogenesis of heart failure and the development of acute decompensation in patients with stable chronic heart failure. This study aimed to compare arterial stiffness indices in patients with heart failure with reduced ejection fraction (HFrEF) during the acute decompensated state, and three months later after hospital discharge during the compensated state.

**Results:**

One hundred patients with acute decompensated HFrEF (NYHA class III and IV) and left ventricular ejection fraction ≤ 35% were included in the study. During the initial and follow-up visits, all patients underwent full medical history taking, clinical examination, transthoracic echocardiography, and non-invasive pulse wave analysis by the Mobil-O-Graph 24-h device for measurement of arterial stiffness. The mean age was 51.6 ± 6.1 years and 80% of the participants were males. There was a significant reduction of the central arterial stiffness indices in patients with HFrEF during the compensated state compared to the decompensated state. During the decompensated state, patients presented with NYHA FC IV (*n* = 64) showed higher AI (24.5 ± 10.0 vs. 16.8 ± 8.6, *p* < 0.001) and pulse wave velocity (9.2 ± 1.3 vs. 8.5 ± 1.2, *p* = 0.021) than patients with NYHA FC III, and despite the relatively smaller number of females, they showed higher stiffness indices than males.

**Conclusions:**

Central arterial stiffness indices in patients with HFrEF were significantly lower in the compensated state than in the decompensated state. Patients with NYHA FC IV and female patients showed higher stiffness indices in their decompensated state of heart failure.

## Background

Arterial stiffness is a state of hardening of arterial walls affecting predominantly the aorta and proximal elastic arteries. This condition is the result of decreased elastic properties of the large elastic arteries, mainly due to age-related progressive degradation of elastin fibers and the deposition of stiff proteins such as collagen in the extracellular matrix (ECM) [1]. It describes the diminished ability of an artery to respond to volume changes by expansion or contraction. Arterial stiffness leads to increased pulse wave velocity (PWV) which, in turn, leads to the early return of reflected waves to the proximal aorta during systole rather than diastole, together with decreased arterial expansion during systole and decreased recoil during diastole will increase the central systolic blood pressure (cSBP), decrease central diastolic blood pressure (cDBP), and increase central pulse pressure (cPP) [2, 3].

Many factors other than age contribute to the development and/or acceleration of arterial stiffness, including genetic abnormalities, diabetes mellitus, arterial hypertension, obesity, chronic kidney disease, the accumulation of advanced glycation end products (AGEs), increased production of reactive oxygen species (ROS), endothelial dysfunction, and endocrinal abnormalities such as increased activity of the renin-angiotensin-aldosterone system (RAAS) and insulin resistance [4–7]. These factors affect arterial stiffness by either ECM modification and/or increasing vascular smooth muscle cell (VSMC) tone [8, 9].

Arterial stiffness is strongly linked to heart failure (HF). Arterial stiffness, as measured by PWV and parameters derived from pulse wave analysis (PWA), was higher in patients with HF than in healthy subjects, and was an independent risk factor for the development of new HF either in normal people or in patients with traditional cardiovascular (CV) risk factors, and was also implicated in the development of acute decompensation of stable patients with chronic HF [10].

The aim of the current study was to compare arterial stiffness parameters during the acute decompensation of heart failure in patients with HFrEF to their compensated state after three months of hospital discharge.

## Methods

### Study design and population

This was a prospective, observational, cohort study conducted over a period of 26 months, from July 1st, 2017, to August 31st, 2019.

The study was approved by the institute’s ethical committee on human research and was given an approval number (I-121015). Informed consent was obtained from all participants.

We studied a convenience sample of patients who were consecutively recruited on the basis of arrival to the hospital with decompensated HF. The included patients were those who were admitted to the hospital with decompensated HFrEF (NYHA functional class III and IV), aged from forty to sixty years, and having an ejection fraction (EF) of ≤ 35%. We excluded patients with severe aortic or mitral valve stenosis or incompetence, significant arrhythmia interfering with accurate PWA, recent myocardial infarction (within two months before admission), estimated glomerular filtration rate (eGFR) < 60 ml/min/1.73 m^2^ of body surface area, patients who received vasopressors or inotropes during hospitalization, and patients with acute de novo HF, e.g., acute myocarditis or on top of acute coronary syndrome (ACS). To ensure an adequate period with a stable compensated state for each patient, patients who were re-hospitalized due to recurrence of decompensation during the follow-up period (3 months) were also excluded.

All patients were studied at baseline during hospital admission with decompensated HF and during the follow-up visit three months after hospital discharge provided that they remained in the compensated state.

### Baseline clinical evaluation

#### Medical history taking

Age, gender, NYHA class, and history of cardiovascular risk factors (hypertension, DM, smoking).

#### Clinical examination

Patients were examined specifically for signs of HF; elevated jugular venous pressure, bibasilar inspiratory crackles, lower limb edema, and S3 gallop.

#### Electrocardiogram (ECG)

An ECG was done for the detection of arrhythmias or any other abnormalities.

#### Routine labs

Complete blood count (CBC), renal function tests (serum creatinine and urea), and serum electrolytes (sodium and potassium) were done for all patients.

#### Transthoracic echocardiography (TTE)

Transthoracic echocardiography (TTE) was performed using the Philips Affiniti 70 ultrasound machine, equipped with a 2.5 MHz transducer. The standard echocardiographic views, obtained from the parasternal, apical, and subcostal windows, were done according to the recommendations of the American Society of Echocardiography (ASE) [11]. A 2D-guided M-mode was used for the measurement of left ventricular end-diastolic and end-systolic diameters, diastolic interventricular septal and posterior wall thickness, and left atrial diameter. The left ventricular ejection fraction (LVEF) was measured by the biplane modified Simpson's rule. The diastolic function was assessed as follows; peak early diastolic velocity (E wave velocity) and peak late diastolic velocity caused by atrial contraction (A wave velocity), then the E/A ratio was calculated. Tissue Doppler imaging (TDI) was used for the measurement of early (e' wave) and late (a' wave) diastolic velocities of the septal and lateral mitral annulus and the average of septal and lateral mitral annular E/e' ratios were calculated and used as an indicator of LV filling pressure. Pulmonary artery systolic pressure (PASP) was calculated using the simplified Bernoulli equation from the peak tricuspid regurgitation velocity and adding this to an estimate of the right atrial pressure according to the inferior vena cava size and its inspiratory collapsibility.

#### Non-invasive PWA

The Mobil-O-Graph 24 h PWA device was used for PWA [12]. It is an oscillometric blood pressure measurement device that is capable of monitoring and analyzing arterial pulse waves. It was previously validated against other non-invasive devices for PWA. The measurements were conducted in a quiet room, while the patient was in the supine or semi-sitting position. Appropriate cuff size was selected according to the arm circumference. The cuff was applied around the patient’s arm, keeping its lower border 2 cm above the cubital fossa and the tube connecting the cuff to the device projecting upwards. Two blood pressure measurements were done, spaced 1–2 min apart in each arm, and if there was a difference of more than 10 mmHg between both arms, the arm with the higher reading was used for the PWA. The device was manually programmed to measure and record pulse waves ten times over thirty minutes. At each time of measurement, the cuff was kept inflated at the level of the diastolic blood pressure in the brachial artery for 10 s for PWA. After finishing all measurements, the cuff was removed from around the patient's arm and the device was connected to a special software program (HMS-Client Server Hypertension Management Software) installed on the computer using the Bluetooth technique for the retrieval of the measured data.

The ARCSOlver algorithm implemented in the Mobil-O-Graph 24 h PWA device reconstructed the central pulse wave by applying a general transfer function on the recorded brachial arterial pulse waves [13, 14]. The PWA was carried on the reconstructed central pulse wave to derive parameters of arterial stiffness; cSBP, cDBP, cPP, augmentation pressure (AP), augmentation index (AIx), reflection magnitude (RM), and PWV. The AIx is affected by some variables, especially the heart rate; so, an index normalized for a heart rate of 75 beats/min (AIx@75) was used [15]. The PWV is defined as the velocity with which the pulse wave travels along the arterial tree and is measured in meters per second (normally < 10 m/s) [16].

### Follow-up

Follow-up visits were scheduled three months after hospital discharge so long as patients were still in the compensated state. During the follow-up visits, patients were clinically assessed and PWA measurements were carried out.

### Primary objective

To detect the possible differences in arterial stiffness parameters in the decompensated versus the compensated state of HFrEF.

### Statistical analysis

The analysis of the data collected was done using Statistical Package for Social Sciences version 26 (SPSS 26) software program. All graphs and tables were obtained from the SPSS program. The mean and the standard deviation (SD) were used to describe continuous variables. Frequencies and percentages were used to describe categorical variables. The paired-sample t-test was used to compare the means of continuous variables in the follow-up visit versus the baseline visit, while the independent sample t-test was used to compare the values of two different groups. The Chi-square/Fischer Exact test was used to compare categorical variables. Pearson’s correlation coefficient was used for correlation between continuous variables. *P* values of ≤ 0.05 were considered statistically significant in this study.

## Results

Throughout the study period, from July 1st, 2017, to August 31st, 2019, 100 patients presenting with decompensated HFrEF met the inclusion criteria and were recruited in the study.

### Baseline demographic characteristics

Table 1 summarizes the baseline demographic characteristics of the study participants. The ages of the participants ranged from 40 to 60 years, with a mean value of 51.6 ± 6.1 years. The mean body mass index (BMI) was 30.5 ± 4.9 kg/m^2^, and the participants’ BMI values ranged from 20.8 to 42 kg/m^2^. Fifty-six patients (56%) were obese (BMI ≥ 30 kg/m^2^). Fifty-six patients (56%) participants had ischemic cardiomyopathy (ICM), while the rest of the participants had idiopathic dilated cardiomyopathy (DCM).

**Table 1:** Baseline demographic characteristics of all patients

Age (years)	51.6 ± 6.1
Male patients	80 (80)
BMI (Kg/m^2^)	30.5 ± 4.9
Smokers	51 (51)
DM	64 (64)
Hypertension	46 (46)

Data are presented as number (%) or mean ± SD

BMI, body mass index; DM, diabetes mellitus

### Comparison of the clinical data during the decompensated and compensated states

During the initial enrolment of patients with decompensated HF, 64 (64%) patients presented with NYHA class IV HF, and the remaining patients had NYHA class III HF. During the follow-up visit, while the patients were compensated, 61 (61%) of them had NYHA class II HF, and the remaining patients had NYHA class I HF.

As can be seen in Table 2, arterial stiffness parameters, as represented by the AP, AIx@75, RM, and PWV, significantly decreased in the compensated state as compared to the decompensated state. The cSBP and cDBP showed a mild, yet statistically significant, decrease without significant changes in the cPP, or the peripheral SBP or DBP. In addition, the heart rate significantly decreased in the compensated state.

**Table 2:** Comparison of the clinical data of all patients during the decompensated and compensated states

Clinical data	Decompensated state	Compensated state	*P* value
HR (b/min)	91.7 ± 13.4	75.3 ± 6.9	**< 0.001**
pSBP (mmHg)	114.3 ± 19.6	112.7 ± 12.4	0.158
pDBP (mmHg)	71.6 ± 11.2	70.6 ± 8.0	0.125
pPP (mmHg)	42.7 ± 14.3	42.0 ± 9.9	0.528
cSBP (mmHg)	103 ± 18.4	100.3 ± 12.4	**0.011**
cDBP (mmHg)	71.8 ± 11.4	69.9 ± 7.9	**0.007**
cPP (mmHg)	30.7 ± 12.4	30.4 ± 9.0	0.705
AP (mmHg)	12.5 ± 7.8	6.3 ± 4.1	**< 0.001**
AIx@75 (%)	21.7 ± 10.2	14.1 ± 5.9	**< 0.001**
RM (%)	59.0 ± 9.3	45.5 ± 9.2	**< 0.001**
PWV (m/sec)	8.9 ± 1.3	8.0 ± 1.3	**< 0.001**
LVEDD (cm)	6.8 ± 0.4	6.7 ± 0.4	0.367
LVESD (cm)	5.2 ± 0.5	5.2 ± 0.6	0.052
EF (%)	27.6 ± 3.5	28.0 ± 3.7	**0.005**
LA diameter (cm)	5.0 ± 0.4	4.9 ± 0.4	**0.01**
Mitral annular E/e'	18.9 ± 4.1	15.7 ± 3.0	**< 0.001**
EPASP (mmHg)	46.3 ± 9.1	44.8 ± 7.9	**0.001**
Hb (g/dl)	12.2 ± 2.2	12.4 ± 1.9	**0.007**
Creatinine (mg/dl)	0.9 ± 0.2	0.8 ± 0.1	**< 0.001**
Na (mEq/L)	129.4 ± 5.5	130.8 ± 5.1	**0.002**
K (mEq/L)	4.3 ± 0.8	4.2 ± 0.6	0.581

Significant *P* value (<0.05) is presented in bold

Data are presented as mean ± SD

AIx@75, augmentation index corrected to heart rate of 75 beats per minute; AP, augmentation pressure; cDBP, central diastolic blood pressure; cPP, central pulse pressure; cSBP, central systolic blood pressure; EF, ejection fraction; EPASP, estimated pulmonary artery systolic pressure; Hb, haemoglobin; HR, heart rate; LA, left atrium; LVEDD, left ventricular end-diastolic diameter; LVESD, left ventricular end-systolic diameter; LVH, left ventricular hypertrophy; pDBP, peripheral diastolic blood pressure; pPP, peripheral pulse pressure; pSBP, peripheral systolic blood pressure; PWV, pulse wave velocity; RM, reflection magnitude; RV, right ventricle; RWMAs, regional wall motion abnormalities

In the compensated state, there was a little (yet statistically significant) increase in the hemoglobin level, serum sodium level, and LVEF and a little statistically significant decrease in the serum creatinine level, mitral annular *E*/*e*', and estimated PASP as compared to the decompensated state. In addition, there was a statistically significant reduction of the left atrial diameter without significant changes in the left ventricular end-diastolic or end-systolic diameters.

### Subgroup analysis

#### Comparison between patients with NYHA class IV HF and those with NYHA class III

Patients with decompensated HF NYHA class IV (*n* = 64), compared to those with NYHA class III (*n* = 36), Table 3, had a significantly higher cPP, AP, AIx@75, and PWV. There were no statistically significant differences in the peripheral blood pressure measurements between the two groups.

**Table 3:** Comparison between baseline PWA in patients with NYHA class III and those with NYHA class IV Heart Failure

Measured parameters by the device	NYHA class III (*n* = 36)	NYHA class IV (*n* = 64)	*P* value
HR (b/min)	89.0 ± 13.2	93.2 ± 13.4	0.141
pSBP (mmHg)	110.7 ± 16.7	116.4 ± 21.0	0.141
pDBP (mmHg)	70.9 ± 11.9	72.0 ± 10.8	0.652
pPP (mmHg)	39.7 ± 12.0	44.3 ± 15.2	0.122
cSBP (mmHg)	99.2 ± 14.6	105.2 ± 20.0	0.089
cDBP (mmHg)	71.5 ± 12.1	72.0 ± 11.1	0.829
cPP (mmHg)	27.4 ± 9.5	32.6 ± 13.4	**0.027**
AP (mmHg)	7.8 ± 5.7	15.2 ± 7.7	**< 0.001**
AIx@75 (%)	16.8 ± 8.6	24.5 ± 10.0	**< 0.001**
RM (%)	59.9 ± 7.7	58.4 ± 10.0	0.448
PWV (m/sec)	8.5 ± 1.2	9.2 ± 1.3	**0.021**

Significant *P* value (<0.05) is presented in bold

Data are presented as mean ± SD

AIx@75, augmentation index corrected to heart rate of 75 beats per minute; AP, augmentation pressure; cDBP, central diastolic blood pressure; cPP, central pulse pressure; cSBP, central systolic blood pressure; HR, heart rate; pDBP, peripheral diastolic blood pressure; pPP, peripheral pulse pressure; pSBP, peripheral systolic blood pressure; PWV, pulse wave velocity; RM, reflection magnitude

#### Comparison between males and females

More females presented with NYHA FC IV HF (75% vs. 61.3%, *p* = 0.252; respectively) and were more likely to present with anemia (10.6 ± 1.6 vs. 12.7 ± 2.1 g/dl, *p* < 0.001; respectively) than males. Males had a significantly lower heart rate, central and peripheral SBP, central and peripheral PP, and AIx@75 compared to females during the decompensated state Table 4. However, in the compensated state, only the heart rate and the augmentation index were significantly lower in males. There was no significant difference in PWV between males and females in both HF states.

**Table 4:** Comparison between males and females during decompensated and compensated states of heart failure

	Decompensated state			Compensated state		
Measured parameters by the device	Males (*n* = 80)	Females (*n* = 20)	*P* value	Males (*n* = 80)	Females (*n* = 20)	*P* value
Heart rate (b/min)	89.8 ± 13.2	99.2 ± 11.7	**0.005**	74.2 ± 6.6	79.7 ± 6.4	**0.001**
Peripheral SBP (mmHg)	112 ± 18.4	123.8 ± 22.2	**0.016**	112.5 ± 12.2	113.7 ± 13.7	0.693
Peripheral DBP (mmHg)	71.3 ± 10.6	72.9 ± 13.2	0.565	70.5 ± 8.1	71.3 ± 8.0	0.683
Peripheral PP (mmHg)	40.6 ± 14.1	50.8 ± 12.3	**0.004**	42.0 ± 10.2	42.4 ± 8.9	0.869
Central SBP (mmHg)	101.2 ± 17.2	110.5 ± 21.5	**0.044**	99.9 ± 12.1	102.3 ± 14.0	0.438
Central DBP (mmHg)	71.5 ± 10.9	73.2 ± 13.5	0.561	70.0 ± 7.9	69.7 ± 8.3	0.851
Central PP (mmHg)	29.2 ± 12.4	36.8 ± 10.6	**0.014**	29.9 ± 9.2	32.7 ± 8.4	0.218
Augmentation pressure (mmHg)	12.1 ± 7.9	14.1 ± 7.7	0.335	6.0 ± 4.2	7.7 ± 3.6	0.097
Augmentation index@75 (%)	19.6 ± 8.1	30.1 ± 13.2	**0.003**	12.8 ± 4.8	19.6 ± 7.2	**0.001**
Reflection magnitude (%)	58.9 ± 8.1	59.4 ± 13.4	0.881	46.0 ± 8.7	43.6 ± 11.2	0.296
Pulse wave velocity (m/s)	8.9 ± 1.3	8.9 ± 1.2	0.799	8.1 ± 1.4	7.9 ± 1.1	0.586

Significant *P* value (<0.05) is presented in bold

Data are presented as mean ± SD

DBP, diastolic blood pressure; PP, pulse pressure; SBP, systolic blood pressure

#### Comparison between patients with ICM and patients with DCM

Patients with ICM had a significantly higher central and peripheral SBP, central and peripheral DBP, and PWV in comparison with patients with DCM, both in the decompensated and compensated states of HF, Table 5. The two groups had comparable results regarding the other arterial stiffness parameters.

**Table 5:** Comparison between Patients with ICM and Patients with DCM in the decompensated and compensated states

Variables	Decompensated state			Compensated state		
	ICM (*n* = 56)	DCM (*n* = 44)	*P* value	ICM (*n* = 56)	DCM (*n* = 44)	*P* value
Heart rate (b/min)	89.7 ± 13.1	94.2 ± 13.5	0.094	73.4 ± 7.0	77.7 ± 6.1	**0.002**
Peripheral SBP (mmHg)	118.6 ± 17.6	108.8 ± 20.9	**0.012**	117.3 ± 9.9	106.8 ± 12.9	**< 0.001**
Peripheral DBP (mmHg)	74.2 ± 9.5	68.3 ± 12.3	**0.012**	72.5 ± 8.2	68.2 ± 7.2	**0.007**
Peripheral PP (mmHg)	44.4 ± 15.4	40.4 ± 12.5	0.164	44.8 ± 10.1	38.6 ± 8.6	**0.002**
Central SBP (mmHg)	107.6 ± 15.1	97.2 ± 20.7	**0.007**	104.6 ± 10.5	95.0 ± 12.8	**< 0.001**
Central DBP (mmHg)	74.6 ± 9.6	68.3 ± 12.6	**0.008**	71.9 ± 8.1	67.4 ± 7.1	**0.004**
Central PP (mmHg)	32.8 ± 13.4	28.0 ± 10.4	0.054	32.6 ± 9.1	27.6 ± 8.2	**0.005**
Augmentation pressure(mmHg)	12.0 ± 7.2	13.2 ± 8.7	0.463	6.0 ± 3.1	6.8 ± 5.1	0.36
Augmentation index@75 (%)	20.4 ± 7.2	23.3 ± 12.9	0.189	13.3 ± 4.8	15.3 ± 7.0	0.96
Reflection magnitude (%)	59.2 ± 8.5	58.7 ± 10.3	0.799	45.9 ± 8.0	45.0 ± 10.7	0.64
Pulse wave velocity (m/sec)	9.2 ± 1.1	8.6 ± 1.4	**0.042**	8.4 ± 1.2	7.7 ± 1.4	**0.014**

Significant *P* value (<0.05) is presented in bold

Data are presented as mean ± SD

DBP, diastolic blood pressure; PP, pulse pressure; SBP, systolic blood pressure

#### Correlation of age and BMI with arterial stiffness parameters

**Fig. 1:**
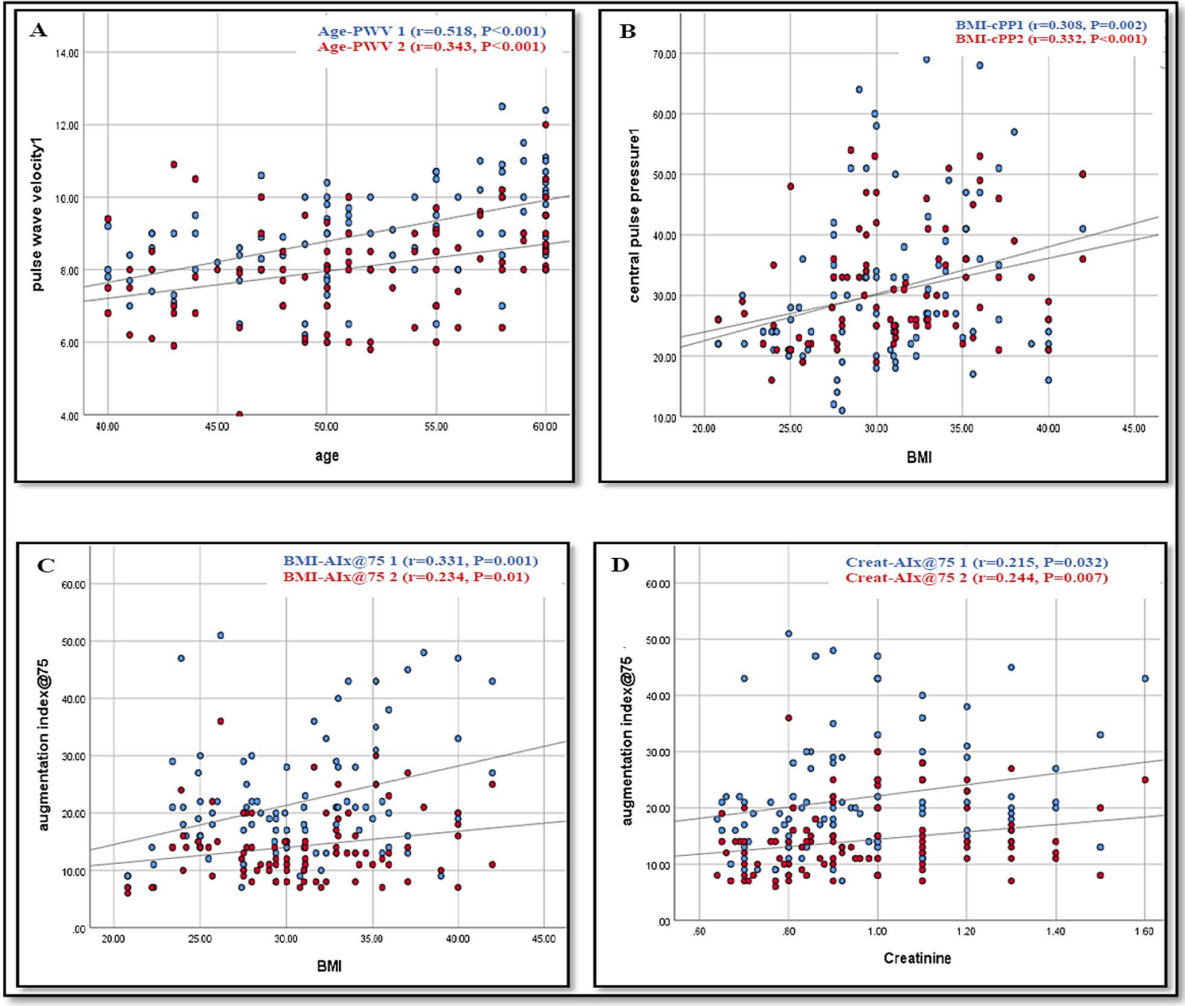
Scatter plots illustrating the correlation of central arterial stiffness parameters with age, body mass index and serum creatinine. BMI, body mass index; PWV, pulse wave velocity; AIx, augmentation index; cPP, central pulse pressure; creat, creatinine. Blue and red dots indicate the correlation during the decompensated and compensated states, respectively. **A** significant positive correlation between age and pulse wave velocity. **B** Significant positive correlation between BMI and central pulse pressure. **C** Significant positive correlation between BMI and AIx@75. **D** Significant positive correlation between serum creatinine and AIx@75

During the decompensated state, there was a moderate significant positive correlation between age and PWV with no significant correlations with other arterial stiffness parameters. On the other hand, there was a weak positive and significant correlation between BMI and all arterial stiffness parameters except for PWV. However, during the compensated state, the age had a significant positive correlation with PWV (*r* = *0.343, p* < *0.001*), while the BMI had significant positive correlations with cPP (*r* = *0.332, p* < *0.001*) and AIx@75 (*r* = *0.234, p* = *0.01*), Figure 1.

## Discussion

Arterial stiffness is an important and independent risk factor for CV diseases [17]. It is strongly linked to the pathogenesis of HF and implicated in the acute decompensation of stable patients with chronic HF [10]. However, there is little data describing the changes in arterial stiffness parameters during the transition from the acute decompensated state of HF to the compensated state. Our study aimed to compare arterial stiffness parameters derived from non-invasive PWA by the Mobil-O-Graph 24 h PWA device in patients with HFrEF during the decompensated state and three months later during the compensated state. The main finding in our study was the significant decrease in arterial stiffness parameters in patients with the compensated state of HFrEF compared to their initial values during the decompensated state. This may be explained by the neurohormonal activation that occurs in decompensated HF, which leads to increased sympathetic tone and peripheral vasoconstriction, resulting in an increased magnitude of wave reflections and PWV [18, 19]. Increased PWV will lead to the early arrival of the reflected waves to the proximal aorta during systole leading to increased AP, cSBP, decreased cDBP, and, finally, increased cPP.

Similar results were reported by Demir et al. [20] who recruited 98 patients (76 males, mean age 59.4 ± 11.6 years) with acute decompensated HFrEF (NYHA class III and IV) and measured the PWV and AIx during the hospitalization then followed the patients up to 18 months with repeated measurements. All patients had ICM with LVEF values of ≤ 35%. They used the Arteriograph for their measurements. During the follow-up period, 70 patients were re-hospitalized for recurrent decompensated HF; of them, 12 patients died during hospitalization. The authors reported that both PWV and AIx were significantly higher in all the 70 patients who were re-hospitalized in comparison with the values obtained when these patients were adequately treated after their initial admission. In addition, they reported that both PWV and AIx were significantly higher in patients who died compared to survivors, and that both were predictors of mortality*.*

Another previous study, in which 80 patients with acute decompensated HF (NYHA class III and IV), was carried out by Sung et al. [21]. The authors measured cfPWV using applanation tonometry and conducted PWA on the carotid pulse wave for the measurement of carotid AP and AIx (which are considered as surrogates for aortic AP and AIx, respectively, in the absence of significant carotid stenosis). The measurements were taken on admission, one day before discharge, and two weeks after discharge, and then, patients were followed up monthly for up to six months. The study endpoints included four CV events (rehospitalization due to decompensation, myocardial infarction, stroke, and death). During the follow-up period, 29 patients experienced CV events. Of the remaining 51 patients that did not experience CV events (42 males, mean age 72.2 ± 14.9 years), 26 patients had HFrEF. However, the authors did not specifically mention the data of the patients with HFrEF but they mentioned the data of the 51 patients, including those with HFpEF. They found that the brachial and central SBP and PP and cfPWV measured two weeks after discharge were less than the admission values in these 51 patients without events but not in those with events who were found to still have high measurements.

On the other hand, Kim et al. [22] studied patients with acute decompensated HF (NYHA class III and IV) during hospitalization and then three months later after discharge while being in the compensated state. The study included 55 patients (25 males, mean age 65.4 ± 12.6 years). During follow-up, seven patients were excluded because they were re-hospitalized within three months after discharge due to recurrent decompensated HF (according to the study’s exclusion criteria), and three patients were lost to follow-up. Of the remaining 45 patients, only 19 had HFrEF (mean EF was 31.8 ± 5.8%). They used applanation tonometry for pressure waves recording at the carotid, radial, femoral, and dorsalis pedis arteries for measurement of the carotid-femoral (central), carotid-radial (upper extremity), and femoral-dorsalis (lower extremity) pulse wave velocities. They reported that the PWV measured at different sites showed no significant changes between the decompensated and compensated states in HFrEF patients. The results of the study by Kim et al. were different from our findings that PWV decreased significantly in transition from the decompensated state to the compensated state. This may be due to the small number of patients included in their study (19 patients), which prevented them from attaining statistical significance during comparative statistics. Also, their patients were obviously older than ours (*mean age was 65.4* ± *12.6 vs. 51.6* ± *6.1 years*) and had a higher prevalence of hypertension (*71% vs. 46%*), which may have influenced the PWV values and the degree of improvement with the transition to the compensated state.

In our study, patients with decompensated HF NYHA class IV had higher cPP, AP, AIx@75, and PWV as compared to those with NYHA class III. This may be explained by the excess neurohormonal activation in NYHA class IV patients, which leads to multiple pathophysiological changes that become more evident with more severe HF. This was supported by data from previous studies by Denardo et al. [23] and Sung et al. [24] that reported increased arterial wave reflection and PWV in patients with acute decompensated HF and their significant increase with more severe HF. Also, as previously mentioned, Sung et al. [21] reported that brachial and central SBP and PP and cfPWV measured 2 weeks after hospital discharge in patients treated for acute decompensated HF decreased progressively in patients without events but not in those with events (including recurrent decompensation) who were still having high measurements. In addition, PWV was shown to have a significant positive correlation with the NT-proBNP level in patients with decompensated HF, which is known to increase with more severe HF [25]. Moreover, Giannattasio et al. [26] showed that arterial stiffness increased with more severe HF and had a positive correlation with impaired cardiac diastolic function.

In our study, male patients, as compared to females, had a significantly lower heart rate, central PP, and AIx@75 in the decompensated state. The higher heart rate in female patients may be due to a higher prevalence of more severe heart failure (NYHA class IV HF) and significantly lower blood hemoglobin levels in comparison with male patients. The higher AIx@75 during the compensated state can be explained by the fact that females generally have higher AP and AIx values than males, partly due to their shorter stature, which makes the reflection sites in the arterial tree closer to the heart and proximal aorta; so, the reflected waves return to the proximal aorta in systole rather than diastole [27]. Previous studies showed gender differences in HF pathophysiology with higher wave reflection, greater pulsatile load, and higher cPP in females than in males [28, 29].

In the current study, we found that patients with ICM had a significantly higher central and peripheral SBP, central and peripheral DBP, and PWV than those with DCM both in the decompensated state and in the compensated state of HF. This may be due to the higher cardiovascular risk profile in ischemic patients. A previous study demonstrated that patients with coronary heart disease have demonstrated increased arterial stiffness, as indicated by higher PWV and AIx values than are found in healthy individuals [30]. Similar to our results, Osmolo vs. kaya et al. [31] found that cfPWV was higher in patients with ICM compared to patients with DCM, while the cPP and AIx did not differ significantly between the two groups. A recent meta-analysis demonstrated that statin therapy could reduce the aortic augmentation index irrespective of the low-density lipoprotein level [32]. Our results showed similar findings where patients who were on regular statin therapy (patients with ICM) demonstrated lower augmentation indexes than patients on no statin therapy (patients with DCM); however, the difference was not statistically significant.

A moderate significant positive correlation was found between age and PWV. Previous studies examined the relationship between age and arterial stiffness as measured by cfPWV, which is the gold standard for measuring arterial stiffness. One of the largest studies is the one by Mattace-Raso et al., which included approximately 18,000 subjects and showed a strong positive correlation with age [33]. Aging leads to the breakdown of elastic fibers in the walls of large elastic arteries and increases the production of stiffer proteins like collagen and proteoglycans. Also, atherosclerosis, endothelial dysfunction, and arterial wall calcification increase with aging. All these factors lead to increased arterial stiffness with increasing age [34].

Similarly, our patients had a significant weak positive correlation between BMI and all parameters of arterial stiffness, except the PWV. In agreement with our results, Rodrigues et al. [35] and Desamericq et al. [36] reported no correlation between the PWV and the BMI, also Biwen et al. [37] reported a significant negative correlation between the brachial-ankle PWV (baPWV) and the BMI. Regarding the AIx, Niruba et al. [38] reported a significant positive correlation with the BMI, while Logan et al. [39] reported a negative correlation between the same two parameters. Some of the mechanisms of increased arterial stiffness in subjects with higher BMI are insulin resistance, the accumulation of AGEs, and increased circulating leptin level. Leptin increases vessel tone, stimulates vascular smooth muscle proliferation, and induces the production of ROS [40, 41].

## Limitations

This was a single-center study, and the number of enrolled patients was relatively small. Females were underrepresented in our study (20%), which may have caused the results to be less representative of the HF patients seen in the daily clinical practice. Because of financial restraints, we could not evaluate the NT-proBNP plasma level to ensure the compensated state of HF during the follow-up visit.

## Conclusions

Arterial stiffness parameters, obtained via non-invasive PWA by the Mobil-O-Graph 24 h PWA device in patients with HFrEF, significantly decreased in the compensated state as compared to the decompensated state of heart failure. Arterial stiffness parameters were higher in patients with more severe HF and patients with ICM. Despite being underrepresented, females showed a higher degree of arterial stiffness than males, both in the decompensated as well as the compensated states. PWV had a positive correlation with age, while other arterial stiffness parameters showed a positive correlation with the BMI.

## Data Availability

The datasets used and/or analyzed during the current study are available from the corresponding author on reasonable request.

## Abbreviations

ACS: Acute coronary syndrome
AGEs: Advanced glycation end products
AIx: Augmentation index
AP: Augmentation pressure
BMI: Body mass index
CBC: Complete blood count
cDBP: Central diastolic blood pressure
cfPWV: Carotid-femoral pulse wave velocity
cPP: Central pulse pressure
cSBP: Central systolic blood pressure
CV: Cardiovascular
DBP: Diastolic blood pressure
DCM: Dilated cardiomyopathy
DM: Diabetes mellitus
ECG: Electrocardiogram
ECM: Extracellular matrix
eGFR: Estimated glomerular filtration rate
FC: Functional class
HF: Heart failure
HFrEF: Heart failure with reduced ejection fraction
ICM: Ischemic cardiomyopathy
LVEF: Left ventricular ejection fraction
NYHA: New York Heart association
PASP: Pulmonary artery systolic pressure
PP: Pulse pressure
PWA: Pulse wave analysis
PWV: Pulse wave velocity
RAAS: Renin angiotensin-aldosterone system
RM: Reflection magnitude
ROS: Reactive oxygen species
SBP: Systolic blood pressure
SD: Standard deviation
TDI: Tissue Doppler imaging
TTE: Transthoracic echocardiography
VSMC: Vascular smooth muscle cell

## References

[1] Tsamis A, Krawiec JT, Vorp DA (2013). Elastin and collagen fibre microstructure of the human aorta in ageing and disease: a review. J R Soc Interface.

[2] Alvim RdO, Santos PCJL, Bortolotto LA, Mill JG, Pereira AdC. Arterial stiffness: pathophysiological and genetic aspects. J Int J Cardiovasc Sci. 2017;30:433–41.

[3] Kim HL, Kim SH (2019). Pulse wave velocity in atherosclerosis. Front Cardiovasc Med.

[4] Wang M, Monticone RE, McGraw KR (2018). Proinflammatory arterial stiffness syndrome: a signature of large arterial aging. J Vasc Res.

[5] Townsend RR (2019). Arterial Stiffness in CKD: A Review. Am J Kidney Dis.

[6] Kuzuya M, Asai T, Kanda S, Maeda K, Cheng XW, Iguchi A (2001). Glycation cross-links inhibit matrix metalloproteinase-2 activation in vascular smooth muscle cells cultured on collagen lattice. Diabetologia.

[7] Rojas A, Romay S, González D, Herrera B, Delgado R, Otero K (2000). Regulation of endothelial nitric oxide synthase expression by albumin-derived advanced glycosylation end products. Circ Res.

[8] Zhao Y, Vanhoutte PM, Leung SW (2015). Vascular nitric oxide: Beyond eNOS. J Pharmacol Sci.

[9] Galis ZS, Khatri JJ (2002). Matrix metalloproteinases in vascular remodeling and atherogenesis: the good, the bad, and the ugly. Circ Res.

[10] Tsao CW, Lyass A, Larson MG, Levy D, Hamburg NM, Vita JA, et al. Relation of Central Arterial Stiffness to Incident Heart Failure in the Community. J Am Heart Assoc. 2015;4(11). 10.1161/JAHA.115.002189 PMC4845230 26597152

[11] Lang RM, Badano LP, Mor-Avi V, Afilalo J, Armstrong A, Ernande L (2015). Recommendations for cardiac chamber quantification by echocardiography in adults: an update from the American Society of Echocardiography and the European Association of Cardiovascular Imaging. J Am Soc Echocardiogr Off Publ Am Soc Echocardiogr.

[12] Weber T, Wassertheurer S, Rammer M, Maurer E, Hametner B, Mayer CC (2011). Validation of a brachial cuff-based method for estimating central systolic blood pressure. Hypertension.

[13] Miyashita H (2012). Clinical assessment of central blood pressure. Curr Hypertens Rev.

[14] Nunan D, Wassertheurer S, Lasserson D, Hametner B, Fleming S, Ward A (2012). Assessment of central haemomodynamics from a brachial cuff in a community setting. BMC Cardiovasc Disord.

[15] Fantin F, Mattocks A, Bulpitt CJ, Banya W, Rajkumar C (2007). Is augmentation index a good measure of vascular stiffness in the elderly?. Age Ageing.

[16] Mancia G, Fagard R, Narkiewicz K, Redon J, Zanchetti A, Bohm M (2013). 2013 ESH/ESC guidelines for the management of arterial hypertension: the Task Force for the Management of Arterial Hypertension of the European Society of Hypertension (ESH) and of the European Society of Cardiology (ESC). Eur Heart J.

[17] Okamoto M, Nakamura F, Musha T, Kobayashi Y (2016). Association between novel arterial stiffness indices and risk factors of cardiovascular disease. BMC Cardiovasc Disord.

[18] Ikonomidis I, Aboyans V, Blacher J, Brodmann M, Brutsaert DL, Chirinos JA, et al. The role of ventricular-arterial coupling in cardiac disease and heart failure: assessment, clinical implications and therapeutic interventions. A consensus document of the European Society of Cardiology Working Group on Aorta & Peripheral Vascular Diseases, European Association of Cardiovascular Imaging, and Heart Failure Association. Eur J Heart Fail. 2019;21(4):402–24. 10.1002/ejhf.1436 30859669

[19] Nardone M, Floras JS, Millar PJ (2020). Sympathetic neural modulation of arterial stiffness in humans. Am J Physiol Heart Circ Physiol.

[20] Demir S, Akpınar O, Akkus O, Nas K, Unal I, Molnar F (2013). The prognostic value of arterial stiffness in systolic heart failure. Cardiol J.

[21] Sung SH, Yu WC, Cheng HM, Chuang SY, Wang KL, Huang CM (2011). Pulsatile hemodynamics and clinical outcomes in acute heart failure. Am J Hypertens.

[22] Kim DB, Baek SH, Jang SW, Her SH, Shin DI, Park CS (2013). Improvement of arterial stiffness in the transition from acute decompensated heart failure to chronic compensated heart failure. Clin Cardiol.

[23] Denardo SJ, Nandyala R, Freeman GL, Pierce GL, Nichols WW (2010). Pulse wave analysis of the aortic pressure waveform in severe left ventricular systolic dysfunction. Circ Heart Fail.

[24] Sung SH, Yu WC, Cheng HM, Lee CW, Lin MM, Chuang SY (2012). Excessive wave reflections on admission predict post-discharge events in patients hospitalized due to acute heart failure. Eur J Heart Fail.

[25] Feola M, Testa M, Ferreri C, Rosso G, Rossi A, Ruocco G. The analysis of arterial stiffness in heart failure patients in comparison with healthy subjects and patients with cardiovascular risk factors. J Clin Med. 2019;8(10):1721. 10.3390/jcm8101721 PMC6833002 31635248

[26] Giannattasio C, Achilli F, Failla M, Capra A, Vincenzi A, Gentile G, et al. Arterial stiffness in heart failure patients: dependance on diastolic dysfunction and plasma aldosterone levels. Eur Heart J Supplements. 2004;6(suppl_F):F30-F4.

[27] Sibiya MJ, Norton GR, Hodson B, Redelinghuys M, Maseko MJ, Majane OH (2014). Gender-specific contribution of aortic augmentation index to variations in left ventricular mass index in a community sample of African ancestry. Hypertens Res.

[28] Costa-Hong VA, Muela HCS, Macedo TA, Sales ARK, Bortolotto LA (2018). Gender differences of aortic wave reflection and influence of menopause on central blood pressure in patients with arterial hypertension. BMC Cardiovasc Disord.

[29] Seeland U, Demuth I, Regitz-Zagrosek V, Steinhagen-Thiessen E, König M (2020). Sex differences in arterial wave reflection and the role of exogenous and endogenous sex hormones: results of the Berlin Aging Study II. J Hypertens.

[30] Nizamov UI, Bekmetova FM, Khoshimov SU, Shek AB, Kurbanov RD (2017). Study of main arteries stiffness in patients with coronary heart disease depending on prevalence of atherosclerosis. Cor Vasa.

[31] Osmolovskaya Y, Glechan A, Skvortsov A, Research VMJA. P7.12 Arterial stiffness in patients with heart failure of ischemic and non-ischemic aetiology. 2009;3(4):186.

[32] Sahebkar A, Pećin I, Tedeschi-Reiner E, Derosa G, Maffioli P, Reiner Ž (2016). Effects of statin therapy on augmentation index as a measure of arterial stiffness: a systematic review and meta-analysis. Int J Cardiol.

[33] Reference Values for Arterial Stiffness C. Determinants of pulse wave velocity in healthy people and in the presence of cardiovascular risk factors: 'establishing normal and reference values'. Eur Heart J. 2010;31(19):2338–50. 10.1093/eurheartj/ehq165 PMC2948201 20530030

[34] Fleenor BS (2013). Large elastic artery stiffness with aging: novel translational mechanisms and interventions. Aging Dis.

[35] Rodrigues SL, Baldo MP, Lani L, Nogueira L, Mill JG, Sa CR (2012). Body mass index is not independently associated with increased aortic stiffness in a Brazilian population. Am J Hypertens.

[36] Desamericq G, Tissot CM, Akakpo S, Tropeano AI, Millasseau S, Macquin-Mavier I (2015). Carotid-femoral pulse wave velocity is not increased in obesity. Am J Hypertens.

[37] Tang B, Luo F, Zhao J, Ma J, Tan I, Butlin M (2020). Relationship between body mass index and arterial stiffness in a health assessment Chinese population. Medicine.

[38] Niruba R., T. Kannan Kumar, Subha K. C., Vijiyalakshmi. Correlation between BMI and arterial stiffness in middle aged subjects. International Journal of Current Research and Review. 2016;8(6).

[39] Logan JG, Kang H, Kim S, Duprez D, Kwon Y, Jacobs DR (2020). Association of obesity with arterial stiffness: the Multi-Ethnic Study of Atherosclerosis (MESA). Vasc Med (London, England).

[40] Jesmin S, Sakuma I, Hattori Y, Kitabatake A (2003). Role of angiotensin II in altered expression of molecules responsible for coronary matrix remodeling in insulin-resistant diabetic rats. Arterioscler Thromb Vasc Biol.

[41] Schäfer K, Halle M, Goeschen C, Dellas C, Pynn M, Loskutoff DJ (2004). Leptin promotes vascular remodeling and neointimal growth in mice. Arterioscler Thromb Vasc Biol.

